# Supplementary material for: Impact of light–matter coupling strength on the efficiency of microcavity OLEDs: a unified quantum master equation approach
Source: Mater Horiz. 2026 Jan 23;13(7):3343–54. doi: 10.1039/d5mh01958c (PMC12848711; doi:10.1039/d5mh01958c)
Supplement: MH-013-D5MH01958C-s001 [file MH-013-D5MH01958C-s001.pdf]

Supplementary Information (SI) for Materials Horizons.
This journal is © The Royal Society of Chemistry 2026

Supplementary information: Impact of light-matter coupling strength on the efficiency of microcavity OLEDs: A unified quantum master equation approach

O. Siltanen,[1,*] K. Luoma,[2] and K. S. Daskalakis[1]

[1] *Department of Mechanical and Materials Engineering, University of Turku, Turku, Finland.*
[2] *Department of Physics and Astronomy, University of Turku, Turku, Finland.*

(Dated: December 9, 2025)

Contents

Supplementary note 1. Vibrational FGR calculations.

Supplementary note 2. Truncating the ISC and RISC rates.

Supplementary note 3. Optical FGR calculations.

Supplementary note 4. Decoherence function and pure-dephasing rate.

Supplementary figure 1. Fitting to the decoherence function.

Supplementary note 5. Omitting unitary dynamics and dephasing.

Supplementary figure 2. Exact open-system dynamics.

Supplementary note 6. Analytical IQE in the weak- and no-coupling regimes.

Supplementary figure 3. IQE in the weak-coupling regime.

Supplementary figure 4. IQE as a function of cut-off energies.

---

* olmisi@utu.fi

SUPPLEMENTARY NOTE 1: VIBRATIONAL FGR CALCULATIONS

Here we present a detailed calculation of the transition rate from $|P_+(\mathbf{k}_\parallel)\rangle$ to $|D_k(\mathbf{k}_\parallel)\rangle$. All the other phonon-mediated transition rates can be calculated similarly.

First, we identify the relevant coupling term in H_I by writing the term $|S_n\rangle\langle S_n|$ in the polariton basis. That is, singlet dephasing causes polariton transitions. Because moving from $|P_+(\mathbf{k}_\parallel)\rangle$ to $|D_k(\mathbf{k}_\parallel)\rangle$ means *creating* a phonon of the energy $E_+(\mathbf{k}_\parallel) - E_s$, we can omit the annihilation operators $\hat{b}_{n,l}$. We also omit multiphonon processes. Thus, the relevant coupling term is $\sum_n \tilde{\sigma}^s_{l'} \frac{\alpha(\mathbf{k}_\parallel)e^{-i\frac{2\pi nk}{N}}}{N} |D_k(\mathbf{k}_\parallel)\rangle\langle P_+(\mathbf{k}_\parallel)|\hat{b}^\dagger_{n,l'}$. l' labels the required transition energy, and we have dropped the label n from $\tilde{\sigma}^s_{n,l}$, because we assume equal coupling strengths at all molecular sites. The initial system-environment eigenstate is, omitting the electromagnetic environment, $|P_+(\mathbf{k}_\parallel)\rangle \otimes |n_{N-k,l'}\rangle$, and the final eigenstate is $|D_k(\mathbf{k}_\parallel)\rangle \otimes |n_{N-k,l'} + 1\rangle$. Here, $N-k$ labels the momentum; note that it too is conserved in the transition. Substituting to FGR, we get

$$\Gamma_{P_+(\mathbf{k}_\parallel)\to D_k(\mathbf{k}_\parallel)} = \frac{2\pi}{\hbar}\Big|\langle D_k(\mathbf{k}_\parallel)| \otimes \langle n_{N-k,l'}+1| \sum_n \tilde{\sigma}^s_{l'} \frac{\alpha(\mathbf{k}_\parallel)e^{-i\frac{2\pi nk}{N}}}{N} |D_k(\mathbf{k}_\parallel)\rangle\langle P_+(\mathbf{k}_\parallel)|\hat{b}^\dagger_{n,l'}|P_+(\mathbf{k}_\parallel)\rangle \otimes |n_{N-k,l'}\rangle\Big|^2 \rho(E_{if}). \tag{S1}$$

Next, we rearrange some terms and switch to the momentum domain,

$$\Gamma_{P_+(\mathbf{k}_\parallel)\to D_k(\mathbf{k}_\parallel)} = \frac{2\pi|\alpha(\mathbf{k}_\parallel)|^2}{\hbar N^2}\Big|\sum_n \tilde{\sigma}^s_{l'} e^{-i\frac{2\pi nk}{N}} \langle n_{N-k,l'}+1|\hat{b}^\dagger_{n,l'}|n_{N-k,l'}\rangle\Big|^2 \rho(E_{if}) \tag{S2}$$

$$= \frac{2\pi|\alpha(\mathbf{k}_\parallel)|^2}{\hbar N^2}\Big|\sum_n \tilde{\sigma}^s_{l'} e^{-i\frac{2\pi nk}{N}} \langle n_{N-k,l'}+1|\frac{1}{\sqrt{N}}\sum_{h=1}^{N} e^{-i\frac{2\pi nh}{N}}\hat{b}^\dagger_{h,l'}|n_{N-k,l'}\rangle\Big|^2 \rho(E_{if}) \tag{S3}$$

$$= \frac{2\pi|\alpha(\mathbf{k}_\parallel)|^2}{\hbar N^3}\Big|\sum_n \tilde{\sigma}^s_{l'} \langle n_{N-k,l'}+1|\Big(\hat{b}^\dagger_{N-k,l'} + \sum_{h\neq N-k} e^{-i\frac{2\pi n(k+h)}{N}}\hat{b}^\dagger_{h,l'}\Big)|n_{N-k,l'}\rangle\Big|^2 \rho(E_{if}) \tag{S4}$$

$$= \frac{2\pi|\alpha(\mathbf{k}_\parallel)|^2}{\hbar N^3}\Big|\sum_n \tilde{\sigma}^s_{l'} \langle n_{N-k,l'}+1|\hat{b}^\dagger_{N-k,l'}|n_{N-k,l'}\rangle\Big|^2 \rho(E_{if}) \tag{S5}$$

$$= \frac{2\pi|\alpha(\mathbf{k}_\parallel)|^2}{\hbar N^3}\Big|N\tilde{\sigma}^s_{l'}\sqrt{\langle n_{N-k,l'}\rangle + 1}\Big|^2 \rho(E_{if}) \tag{S6}$$

$$= \frac{2\pi|\alpha(\mathbf{k}_\parallel)|^2}{\hbar N}\big|\tilde{\sigma}^s_{l'}\big|^2\big(\langle n_{N-k,l'}\rangle + 1\big)\rho(E_{if}). \tag{S7}$$

Finally, we use the standard definitions $\rho(E_{if}) := \delta(|E_f - E_i| - E_j)$ [3] and $\mathcal{J}(E) := \frac{2\pi}{\hbar}\sum_j |\sigma_j|^2 \delta(E - E_j)$ [53] to obtain

$$\Gamma_{P_+(\mathbf{k}_\parallel)\to D_k(\mathbf{k}_\parallel)} = \frac{|\alpha(\mathbf{k}_\parallel)|^2}{N}\tilde{\mathcal{J}}_s\big(E_+(\mathbf{k}_\parallel) - E_s\big)\big[\big\langle n\big(E_+(\mathbf{k}_\parallel) - E_s\big)\big\rangle + 1\big]. \tag{S8}$$

Transitions between the exciton reservoir and lower polariton are weighted by $|\beta(\mathbf{k}_\parallel)|^2$ instead of $|\alpha(\mathbf{k}_\parallel)|^2$, while transitions between the polaritons are weighted by the product $|\alpha(\mathbf{k}_\parallel)|^2|\beta(\mathbf{k}_\parallel)|^2$. If the system goes up in energy, $\langle n(\bullet)\rangle + 1$ is replaced by $\langle n(\bullet)\rangle$, because such a transition cannot occur spontaneously. If the energy of the system does not change, both $\langle n(0)\rangle + 1$ and $\langle n(0)\rangle$ contribute to the transition rate. If the system relaxes to the global ground state, the factor of $1/N$ becomes 1; the global ground state $|\mathcal{G}\rangle \otimes |0\rangle$ is already an eigenstate and does not contain $1/\sqrt{N}$. Here, we would also replace the Ohmic $\tilde{\mathcal{J}}_s$ with the super-Ohmic $\mathcal{J}_{s(t)}$.

SUPPLEMENTARY NOTE 2: TRUNCATING THE ISC AND RISC RATES

A fully quantum mechanical treatment (in the weak- and no-coupling regimes) would give us the ISC/RISC rates [3]

$$\Gamma_{(R)ISC} = \frac{V_{st}^2}{\hbar}\sqrt{\frac{\pi}{\lambda k_B T}}\sum_{l=0}^{\infty}\frac{S^l}{l!}e^{-S}e^{-\frac{(\lambda+l\hbar\omega+\Delta E)^2}{4\lambda k_B T}}, \tag{S9}$$

where the ls label different vibrational levels, S is the Huang-Rhys parameter quantifying the strength of phonon couplings, and $\hbar\omega$ is the phonon energy. Expanding the expressions, we get

$$\Gamma_{(R)ISC} = \frac{V_{st}^2}{\hbar}\sqrt{\frac{\pi}{\lambda k_B T}}[1 - S + \mathcal{O}(S^2)]\left[e^{-\frac{(\lambda+\Delta E)^2}{4\lambda k_B T}} + Se^{-\frac{(\lambda+\hbar\omega+\Delta E)^2}{4\lambda k_B T}} + \mathcal{O}(S^2)\right]. \tag{S10}$$

Because we are working in the weak system-environment coupling regime, we can only keep the linear terms in S. Hence,

$$\Gamma_{(R)ISC} \approx \frac{V_{st}^2}{\hbar}\sqrt{\frac{\pi}{\lambda k_B T}}\left[(1-S)e^{-\frac{(\lambda+\Delta E)^2}{4\lambda k_B T}} + Se^{-\frac{(\lambda+\hbar\omega+\Delta E)^2}{4\lambda k_B T}}\right]. \tag{S11}$$

The difference to the rate given in the main text is therefore

$$\frac{V_{st}^2}{\hbar}\sqrt{\frac{\pi}{\lambda k_B T}}S\left[e^{-\frac{(\lambda+\hbar\omega+\Delta E)^2}{4\lambda k_B T}} - e^{-\frac{(\lambda+\Delta E)^2}{4\lambda k_B T}}\right]. \tag{S12}$$

Since both S and ω are small, this difference can be omitted. For example, $S = 0.001$ and $\hbar\omega = 20$ meV result in < 1 % difference for both ISC and RISC. In fact, the difference would become even smaller for polaritons due to the $1/N$ scaling.

SUPPLEMENTARY NOTE 3: OPTICAL FGR CALCULATIONS

Here we calculate just the rate of emission to free space, Γ_{free}. Γ_{cavity} is then obtained from Γ_{free} by multiplying it by the Purcell factor, which accounts for the modified density of states in the cavity, and the spectral mismatch factor obtained from the time coarse graining.

Since it is the singlets that are coupled with the free-space modes, the photonic parts of the polaritons vanish in FGR. The excitonic weights follow as previously in Supplementary note 1. Hence, it is enough to examine jumps from $|D_k(\mathbf{k}_\parallel)\rangle$, $k \in [1, N]$, to $|\mathcal{G}\rangle \otimes |0\rangle$. Note that $k = N$ is the momentum index of the polaritons and 0 refers to the number of *cavity* photons.

$$\Gamma_{D_k(\mathbf{k}_\parallel)\to\mathcal{G}\otimes 0} = \frac{2\pi}{\hbar}\Big|\langle\mathcal{G}|\otimes\langle 0|\otimes\langle n_{\mathbf{k}_\parallel}+1|\sum_n f(\mathbf{k}_\parallel)\frac{e^{i\frac{2\pi nk}{N}}}{\sqrt{N}}|\mathcal{G}\rangle\otimes|0\rangle\langle D_k(\mathbf{k}_\parallel)|\hat{c}^\dagger_{\mathbf{k}_\parallel}|D_k(\mathbf{k}_\parallel)\rangle\otimes|n_{\mathbf{k}_\parallel}\rangle\Big|^2\rho(E_{if}) \tag{S13}$$

$$= \frac{2\pi}{\hbar}\frac{|f(\mathbf{k}_\parallel)|^2}{N}\big(\langle n_{\mathbf{k}_\parallel}\rangle + 1\big)\Big|\sum_n e^{i\frac{2\pi nk}{N}}\Big|^2\rho(E_{if}) \tag{S14}$$

$$\approx \frac{2\pi}{\hbar}\frac{|f(\mathbf{k}_\parallel)|^2}{N}\Big|\sum_n \delta_{k,N}\Big|^2\rho(E_{if}) \tag{S15}$$

$$= \frac{2\pi}{\hbar}N|f(\mathbf{k}_\parallel)|^2\rho(E_{if})\delta_{k,N} \tag{S16}$$

$$= \frac{2\pi}{\hbar}N\mu^2\frac{E_\pm(\mathbf{k}_\parallel)}{2\epsilon_0 V_f}\frac{V_f E_\pm(\mathbf{k}_\parallel)^2}{\pi^2\hbar^3c^3}\delta_{k,N} \tag{S17}$$

$$= N\frac{\mu^2 E_\pm(\mathbf{k}_\parallel)^3}{\pi\epsilon_0\hbar^4c^3}\delta_{k,N}. \tag{S18}$$

Here we used the density of states $\rho(E_{if}) = \frac{V_f E_\pm(\mathbf{k}_\parallel)^2}{\pi^2\hbar^3c^3}$ [48]. Furthermore, for the energies we are interested in, $\langle n_{\mathbf{k}_\parallel}\rangle \approx 0$. As already mentioned, the emission rate is still multiplied by the excitonic weight of the emitting polariton. And note that it is only the polaritons that emit; due to destructive interference, the sum over n survives only when $k = N$.

SUPPLEMENTARY NOTE 4: DECOHERENCE FUNCTION AND PURE-DEPHASING RATE

With a free Hamiltonian H_0, interaction Hamiltonian of the form $\sum_X |X\rangle\langle X| \otimes E_X$, and initial environment state $|\xi\rangle$, coherence terms $\langle X|\rho(t)|Y\rangle$ can be generally written as $\langle X|\rho(0)|Y\rangle\kappa_{XY}(t)$, where

$$\kappa_{XY}(t) = \langle\xi_Y(t)|\xi_X(t)\rangle \tag{S19}$$

$$= \langle\xi|e^{i\int_0^t ds e^{iH_0t/\hbar}E_Y e^{-iH_0t/\hbar}/\hbar} e^{-i\int_0^t ds e^{iH_0t/\hbar}E_X e^{-iH_0t/\hbar}/\hbar}|\xi\rangle \tag{S20}$$

is the *decoherence function* [53]. While our interaction Hamiltonian (3) contains cross-terms $|X\rangle\langle Y|$, all couplings are assumed weak, and so we can use Eq. (S20) to estimate the contribution of vibrational fluctuations to the actual decoherence function.

Using the thermal equilibrium state $|\psi_{th}\rangle$ (purification of ρ_{th}) as $|\xi\rangle$, the time-dependent phonon state coupled with nth singlet becomes

$$|\xi_n(t)\rangle = \exp\Big[-\frac{i}{\hbar}\int_0^t dt' \sum_l \tilde{\sigma}^s_{n,l}(e^{-i\omega_{n,l}t'}\hat{b}_{n,l} + e^{i\omega_{n,l}t'}\hat{b}^\dagger_{n,l})\Big]|\psi_{th}\rangle \tag{S21}$$

$$= \exp\Big[-\frac{i}{\hbar}\sum_l \tilde{\sigma}^s_{n,l}\Big(\frac{1-e^{i\omega_{n,l}t}}{\omega_{n,l}}\hat{b}^\dagger_{n,l} - \frac{1-e^{-i\omega_{n,l}t}}{\omega_{n,l}}\hat{b}_{n,l}\Big)\Big]|\psi_{th}\rangle \tag{S22}$$

$$= \prod_l D\Big(\frac{\tilde{\sigma}^s_{n,l}}{\hbar}\frac{1-e^{i\omega_{n,l}t}}{\omega_{n,l}}\Big)|\psi_{th}\rangle. \tag{S23}$$

Here, $D(\bullet)$ is the displacement operator. The phonon state coupled with the ground state is simply $|\psi_{th}\rangle$.

Calculating the inner product $\langle\psi_{th}|\xi_n(t)\rangle$, using the thermal displacement average, and taking the continuum limit, we get

$$\frac{\langle\mathcal{G}|\rho(t)|S_n\rangle}{\langle\mathcal{G}|\rho(0)|S_n\rangle} = \kappa_n(t) = \langle\psi_{th}|\prod_l D\Big(\frac{\tilde{\sigma}^s_{n,l}}{\hbar}\frac{1-e^{i\omega_{n,l}t}}{\omega_{n,l}}\Big)|\psi_{th}\rangle \tag{S24}$$

$$= \exp\Big\{-\sum_l \frac{|\tilde{\sigma}_{n,l}|^2}{\hbar^2\omega^2_{n,l}}\big[1-\cos(\omega_{n,l}t)\big]\coth\Big(\frac{\omega_{n,l}}{2k_BT}\Big)\Big\} \tag{S25}$$

$$\to \exp\Big\{-\frac{\hbar}{2\pi}\int dE\frac{\tilde{\mathcal{J}}_s(E)}{E^2}\Big[1-\cos\Big(\frac{Et}{\hbar}\Big)\Big]\coth\Big(\frac{E}{2\hbar k_BT}\Big)\Big\}. \tag{S26}$$

Fig. S1 shows both a sample of $|\kappa_n(t)|$ and the function $\exp(-\Phi^2t^2)$ fitted to it using a nonlinear least-squares method. The fitting yields the pure-dephasing rate $\Phi \approx 3.105\times10^{13}\ \mathrm{s}^{-1}$, which is used in the main text to determine which coupling regime the system is in.

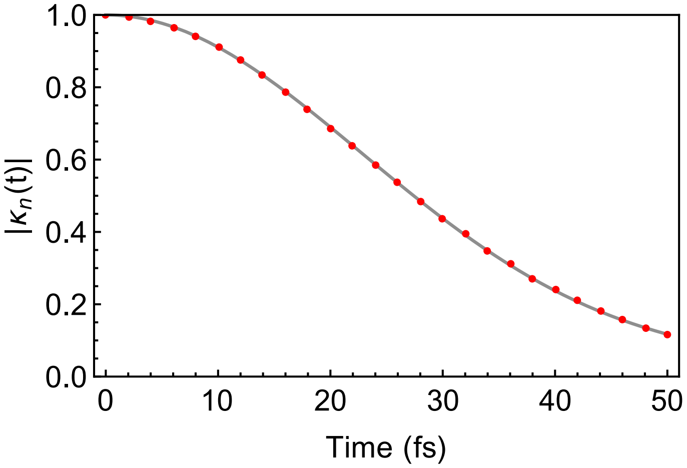

FIG. S1. The decoherence function $|\kappa_n(t)|$ (or the pure-dephasing contribution of it; red dots) and the fitted function $\exp(-\Phi^2t^2)$ (gray curve).

SUPPLEMENTARY NOTE 5: OMITTING UNITARY DYNAMICS AND DEPHASING

Expressing $|S_n\rangle\langle\mathcal{G}|$ in the eigenbasis reveals that electrical pumping creates coherences between the upper and lower polaritons, which we denote by $\rho_{+-}(\mathbf{k}_\parallel, t)$. Thus, the exact ground-state dynamics, with coherent contributions, acquires an extra term,

$$\frac{d}{dt}\langle\mathcal{G}\otimes 0(t)\rangle = \cdots - 2|\alpha(\mathbf{k}_\parallel)||\beta(\mathbf{k}_\parallel)|\kappa(\mathbf{k}_\parallel)\Re\big[\rho_{+-}(\mathbf{k}_\parallel, t)\big], \tag{S27}$$

and the coherences evolve according to

$$\begin{aligned}\frac{d}{dt}\rho_{+-}(\mathbf{k}_\parallel, t) =& |\alpha(\mathbf{k}_\parallel)||\beta(\mathbf{k}_\parallel)|\frac{JA}{4eN}\langle\mathcal{G}\otimes 0(t)\rangle \\ &- \frac{1}{2}\Big[\sum_{X\neq P_+(\mathbf{k}_\parallel)}\Gamma_{P_+(\mathbf{k}_\parallel)\to X} + \sum_{X\neq P_-(\mathbf{k}_\parallel)}\Gamma_{P_-(\mathbf{k}_\parallel)\to X} + \frac{i}{\hbar}2\big(E_+(\mathbf{k}_\parallel) - E_-(\mathbf{k}_\parallel)\big)\Big]\rho_{+-}(\mathbf{k}_\parallel, t).\end{aligned} \tag{S28}$$

Dark-state coherences vanish when summed over n as a result of destructive interference.

We can neglect coherences in the main text for several reasons. 1) Their effective pumping rate is negligible, scaling as $1/N$. 2) The prefactor $|\alpha(\mathbf{k}_\parallel)||\beta(\mathbf{k}_\parallel)|$ implies that coherences are created even less efficiently when moving away from the $E_s = E_c(\mathbf{k}_\parallel)$ resonance. 3) The magnitude of coherences is bounded from above by $|\rho_{+-}(\mathbf{k}_\parallel, t)| \leq \sqrt{\langle P_+(\mathbf{k}_\parallel, t)\rangle\langle P_-(\mathbf{k}_\parallel, t)\rangle}$, and the polariton populations themselves are very small due to their short lifetimes. 4) The coherences directly influence only the ground-state population, so any indirect influence on other states is greatly diminished. 5) Various dephasing mechanisms—though not explicitly considered here in the polariton basis—will further suppress the impact of coherences.

Let us illustrate how negligible the coherences are in the large-N case. Fig. S2 shows the exact population and coherence dynamics of the upper polariton at $\mathbf{k}_\parallel = \mathbf{0}$, when the system is initially in the global ground state $|\mathcal{G}\rangle\otimes|0\rangle$. The magnitude of the coherence is nine orders of magnitude smaller than the corresponding population. In fact, the approximate population used in the main text differs from its exact counterpart by less than 0.001 %.

For the above reasons, while polariton coherences might play a bigger role in single-molecule strong coupling, we can safely ignore coherences and the dephasing that would just destroy them in the large-N case.

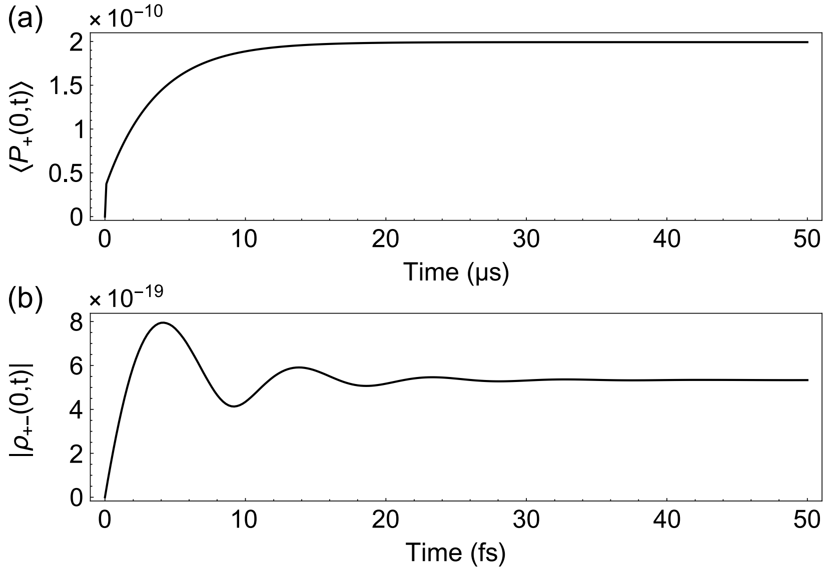

FIG. S2. Exact dynamics of (a) $\langle P_+(0,t)\rangle$ and (b) $|\rho_{+-}(0,t)|$, when $L_c = 150$ nm and $N = 5\times10^5$. All the other parameters are given in Fig. 3(b).

SUPPLEMENTARY NOTE 6: ANALYTICAL IQE IN THE WEAK- AND NO-COUPLING REGIMES

In the weak-coupling regime, it is simpler to evaluate the population dynamics and IQE using the molecular-site basis $\{|S_n\rangle\}_{n=1}^{N}$. With fixed $\mathbf{k}_{\|}$, the system of coupled rate equations reads

$$\frac{d}{dt}\langle S(t)\rangle = \frac{JA}{4e}\langle \mathcal{G}\otimes 0(t)\rangle + \Gamma_{RISC}\langle T(t)\rangle - \big(\Gamma_r + \Gamma_{cavity}(\mathbf{k}_{\|}) + \Gamma_{s,nr} + \Gamma_{ISC}\big)\langle S(t)\rangle \tag{S29}$$

$$\frac{d}{dt}\langle T(t)\rangle = \frac{3JA}{4e}\langle \mathcal{G}\otimes 0(t)\rangle + \Gamma_{ISC}\langle S(t)\rangle - (\Gamma_{t,nr} + \Gamma_{RISC})\langle T(t)\rangle \tag{S30}$$

$$\frac{d}{dt}\langle \mathcal{G}\otimes 1_{\mathbf{k}_{\|}}(t)\rangle = \Gamma_{cavity}(\mathbf{k}_{\|})\langle S(t)\rangle - \kappa(\mathbf{k}_{\|})\langle \mathcal{G}\otimes 1_{\mathbf{k}_{\|}}(t)\rangle \tag{S31}$$

$$\frac{d}{dt}\langle \mathcal{G}\otimes 0(t)\rangle = (\Gamma_r + \Gamma_{s,nr})\langle S(t)\rangle + \Gamma_{t,nr}\langle T(t)\rangle + \kappa(\mathbf{k}_{\|})\langle \mathcal{G}\otimes 1_{\mathbf{k}_{\|}}(t)\rangle - \frac{JA}{e}\langle \mathcal{G}\otimes 0(t)\rangle. \tag{S32}$$

Under steady-state conditions, the IQE can be written as

$$\mathrm{IQE} = \frac{1}{K}\sum_{\mathbf{k}_{\|}} \frac{\Gamma_r\langle S\rangle + \kappa(\mathbf{k}_{\|})\langle \mathcal{G}\otimes 1_{\mathbf{k}_{\|}}\rangle}{JA/e} \times 100\% \tag{S33}$$

$$= \frac{1}{K}\sum_{\mathbf{k}_{\|}} \frac{\big(\Gamma_r + \Gamma_{cavity}(\mathbf{k}_{\|})\big)\langle S\rangle}{JA/e} \times 100\% \tag{S34}$$

$$= \frac{1}{K}\sum_{\mathbf{k}_{\|}} \frac{\big(\Gamma_r + \Gamma_{cavity}(\mathbf{k}_{\|})\big)\big(\Gamma_{RISC} + \frac{1}{4}\Gamma_{t,nr}\big)}{\big(\Gamma_r + \Gamma_{cavity}(\mathbf{k}_{\|}) + \Gamma_{s,nr}\big)\big(\frac{3JA}{4e} + \Gamma_{RISC} + \Gamma_{t,nr}\big) + \frac{JA}{e}\big(\Gamma_{ISC} + \Gamma_{RISC} + \frac{1}{4}\Gamma_{t,nr}\big) + \Gamma_{ISC}\Gamma_{t,nr}} \times 100\%. \tag{S35}$$

Fig. S3 shows the weak-coupling IQE as a function of cavity thickness, when $K = 313$. We reach the maximum IQE of 97.41 % at 143.10 nm, i.e., when the cavity mode at normal incidence is slightly below the singlet resonance.

The no-coupling version of Eq. (S35) is obtained by simply setting $\Gamma_{cavity}(\mathbf{k}_{\|}) = 0\ \forall \mathbf{k}_{\|}$. Physically, this can be achieved by matching the refractive indices of the emitter and mirror materials. For example, the aluminum mirrors considered in this work could be replaced with indium tin oxide. With $\Gamma_{cavity}(\mathbf{k}_{\|}) = 0$, Eq. (S35) yields the reference IQE of 96.87 %. This is also evident in Fig. S3. When the cavity is very thin, the cavity modes are far out of singlet resonance. Assuming too few molecules for polariton formation, the singlets couple only with the free-space modes.

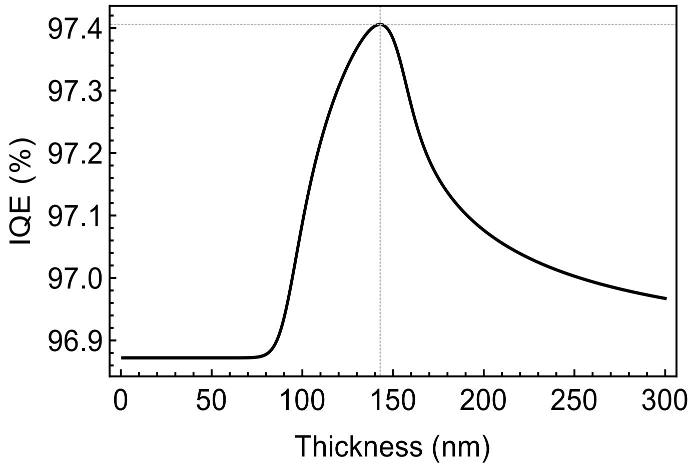

FIG. S3. IQE in the weak-coupling regime, as a function of the cavity thickness. We get the no-coupling IQE at small cavity thicknesses, where there is effectively no coupling due to large spectral mismatch (assuming small N).

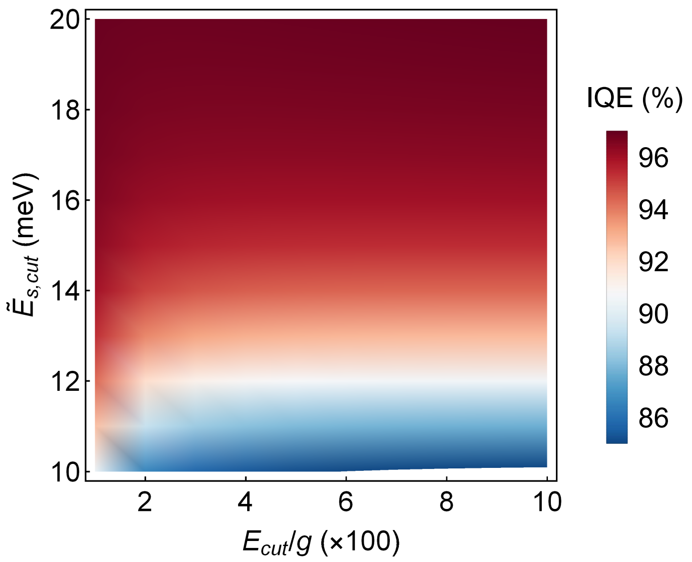

FIG. S4. IQE as a function of the dephasing cut-off $\tilde{E}_{s,cut}$ and the rescaled light-matter coupling cut-off $E_{cut}(\mathbf{k}_\parallel)/g(\mathbf{k}_\parallel)$, when $L_c = 125$ nm and $N = 10^5$. All the other parameters are given in Fig. 3(b). Increasing $\tilde{E}_{s,cut}$ leads to stronger dephasing and thus the polaritons getting populated more efficiently. Increasing $E_{cut}(\mathbf{k}_\parallel)/g(\mathbf{k}_\parallel)$ has the opposite effect, as it widens the intrinsically inefficient strong-coupling regime.
